# Supplementary material for: Knowledge transfer, knowledge gaps, and knowledge silos in citation networks
Source: PLoS One. 2025 Aug 1;20(8):e0329302. doi: 10.1371/journal.pone.0329302 (PMC12316298; doi:10.1371/journal.pone.0329302)
Supplement: S1 Appendix — (PDF) [file pone.0329302.s001.pdf]

S1 Appendix

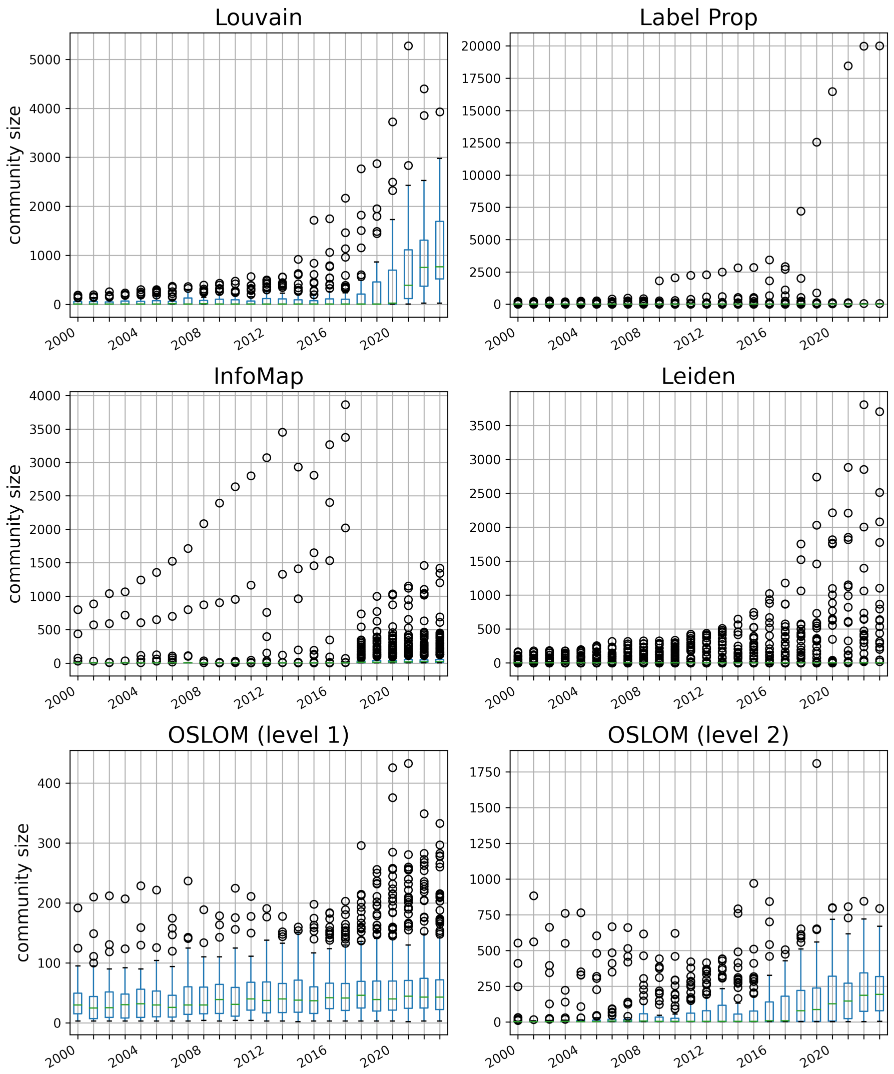

Figure S1: Community size distributions for 5 different community detection algorithms. For OSLOM - a hierarchical community finding method - we include the sizes of communities at the first and second level in the hierarchy.

Figure S1 shows the distribution of community sizes in each time step graph (2000 - 2023), for 5 different community detection methods. According to these distributions, it is apparent that different community detection methods group papers in the citation network very differently. For example, the *LableProp* method groups the majority of papers into a single, very large community, with a long tail of very small communities. Similarly, the *InfoMap* methods shows a bias towards a small number of very large communities. Conversely, the communities discovered by the *Louvain* method appear to grow stably in size as the network grows. At level 2, the *OSLOM* method shows similar behaviour, with the additional benefit that we can consider the subdivisions within these research areas at level 1.
